# Antitumor activity of Z-endoxifen in aromatase inhibitor-sensitive and aromatase inhibitor-resistant estrogen receptor-positive breast cancer

**DOI:** 10.1186/s13058-020-01286-7

**Published:** 2020-05-19

**Authors:** Swaathi Jayaraman, Xiaonan Hou, Mary J. Kuffel, Vera J. Suman, Tanya L. Hoskin, Kathryn E. Reinicke, David G. Monroe, Krishna R. Kalari, Xiaojia Tang, Megan A. Zeldenrust, Jingfei Cheng, Elizabeth S. Bruinsma, Sarah A. Buhrow, Renee M. McGovern, Stephanie L. Safgren, Chad A. Walden, Jodi M. Carter, Joel M. Reid, James N. Ingle, Matthew M. Ames, John R. Hawse, Matthew P. Goetz

**Affiliations:** 1 Department of Oncology, Mayo Clinic, Rochester, MN USA; 2 Department of Health Sciences Research, Mayo Clinic, Rochester, MN USA; 3 Department of Biochemistry and Molecular Biology, Mayo Clinic, Rochester, MN USA; 4 Department of Laboratory Medicine and Pathology, Mayo Clinic, Rochester, MN USA; 5 Department of Molecular Pharmacology and Experimental Therapeutics, Mayo Clinic, Rochester, MN USA

**Keywords:** Z-endoxifen, Tamoxifen, Tumor growth in vivo, Estrogen-regulated genes, AI-resistant and AI-sensitive ER+ breast cancer, Signaling kinase

## Abstract

**Background:**

The tamoxifen metabolite, Z-endoxifen, demonstrated promising antitumor activity in endocrine-resistant estrogen receptor-positive (ER+) breast cancer. We compared the antitumor activity of Z-endoxifen with tamoxifen and letrozole in the letrozole-sensitive MCF7 aromatase expressing model (MCF7AC1), as well as with tamoxifen, fulvestrant, exemestane, and exemestane plus everolimus in a letrozole-resistant MCF7 model (MCF7LR).

**Methods:**

MCF7AC1 tumor-bearing mice were randomized to control (no drug), letrozole (10 μg/day), tamoxifen (500 μg/day), or Z-endoxifen (25 and 75 mg/kg). Treatment in the letrozole arm was continued until resistance developed. MCF7LR tumor-bearing mice were then randomized to Z-endoxifen (50 mg/kg) or tamoxifen for 4 weeks and tumors harvested for microarray and immunohistochemistry analysis. The antitumor activity of Z-endoxifen in the MCF7LR tumors was further compared in a second in vivo study with exemestane, exemestane plus everolimus, and fulvestrant.

**Results:**

In the MCF7AC1 tumors, both Z-endoxifen doses were significantly superior to control and tamoxifen in reducing tumor volumes at 4 weeks. Additionally, the 75 mg/kg Z-endoxifen dose was additionally superior to letrozole. Prolonged letrozole exposure resulted in resistance at 25 weeks. In MCF7LR tumor-bearing mice, Z-endoxifen significantly reduced tumor volumes compared to tamoxifen, letrozole, and exemestane, with no significant differences compared to exemestane plus everolimus and fulvestrant. Additionally, compared to tamoxifen, Z-endoxifen markedly inhibited ERα target genes, Ki67 and Akt expression in vivo.

**Conclusion:**

In endocrine-sensitive and letrozole-resistant breast tumors, Z-endoxifen results in robust antitumor and antiestrogenic activity compared to tamoxifen and aromatase inhibitor monotherapy. These data support the ongoing development of Z-endoxifen.

## Background

The selective estrogen receptor modulator (SERM) tamoxifen is commonly prescribed for prevention of breast cancer and the treatment of early, advanced, and metastatic pre- and postmenopausal estrogen receptor-positive (ER+) breast cancers. Tamoxifen is extensively metabolized in humans by the cytochrome P450 2D6 enzyme, CYP2D6, into potent antiestrogenic metabolites 4-hydroxytamoxifen (4HT) and 4-hydroxy *N*-desmethyl tamoxifen (endoxifen), with pre-clinical studies demonstrating superior antiestrogenic activity of 4HT and endoxifen compared to the parent drug [1–3]. Tamoxifen-treated patients with CYP2D6 genetic polymorphisms exhibit lower endoxifen concentrations, and many reports have demonstrated a higher risk of recurrence [4–6]. Pharmacokinetics studies have shown that direct oral administration of endoxifen yields substantially higher concentrations compared to endoxifen levels achieved via tamoxifen [7, 8]. Based on these data, prospective clinical trials of oral Z-endoxifen (synthesized at the National Cancer Institute, Bethesda, MD) were initiated with initial observations demonstrating substantial Z-endoxifen plasma concentrations (2–6 μM), manageable side effects, and promising antitumor activity in aromatase inhibitor (AI)- and tamoxifen-resistant patients [9].

Given these data, we further characterized the in vivo activity of Z-endoxifen using the aromatase expressing MCF7 cell line model (MCF7AC1). We chose this model given its track record for predicting the clinical efficacy of AI’s over tamoxifen, as well the efficacy of fulvestrant in combination with anastrozole compared to AI monotherapy [10, 11]. In addition, we developed a letrozole-resistant model (MCF7LR) to compare the antitumor activity of Z-endoxifen with tamoxifen, exemestane, exemestane plus everolimus, and fulvestrant and to further characterize the transcriptome of tamoxifen and Z-endoxifen. Finally, we assessed the antitumor activity of Z-endoxifen across a broad panel of ER+, ER-negative (ER−), and human epidermal growth factor receptor 2-positive (HER2+) cell lines.

## Methods

### Cell lines

MCF7 human breast cancer cells stably transfected with the human aromatase gene (MCF7AC1) [12] (a kind gift from Angela H. Brodie, University of Maryland, Baltimore, MD), were cultured in phenol-red-free IMEM medium supplemented with 10% fetal bovine serum (FBS) and 600 μg/ml geneticin (G418). Letrozole-resistant MCF7LR cells were cultured in phenol-red free IMEM medium supplemented with 10% charcoal stripped FBS (Hyclone), 1% antibiotic-antimycotic (AA), 1 μM letrozole, and 1 nM androstenedione.

T47D, MDA-MB-231, MDA-MB-468, BT474, and BT20 cells cultured in a mixture of DMEM and Ham F12 (1:1 ratio) medium supplemented with 10% FBS, 1% AA. Cells were obtained from ATCC. MCF7 cells engineered to overexpress HER2 (MCF7-HER2–18) or the empty vector (MCF7-neo) (a kind gift from Rachel Schiff, Baylor College of Medicine, Houston, TX) were cultured in high glucose DMEM medium supplemented with 10% FBS, 1X glutamax and 400 μg/ml G418. All reagents were from Gibco unless otherwise stated. Cells were authenticated by short-tandem repeat profiling at Genetica Cell Line Testing.

### Cell line xenograft models

MCF7AC1 cells at 2.5 × 10^6^ cells per 100 μl of 1:1 phosphate-buffered saline (PBS) to Matrigel mixture were injected subcutaneously into the right and left flanks of 4–6-week-old ovariectomized BALB/c athymic female nude mice (Harlan Laboratories). Upon development of palpable tumors (tumor volume, ≥ 300 mm^3^), the mice were randomized into treatment groups (*n* = 30 mice/group) and were administered control (no drug), tamoxifen (500 μg/day) or letrozole (10 μg/day) (all prepared as suspensions in 0.3% hydroxypropyl cellulose) subcutaneously or Z-endoxifen (25 mg/kg or 75 mg/kg) (prepared as suspensions in PEG400 to ascorbic acid 50:50) by oral gavage. All animals were supplemented with 1.4-mg 90-day-release estrogen pellets (Innovative Research), and the drugs were administered once daily. Tumor volume was measured weekly using calipers and calculated using the formula: (width^2^ × length)/2 for the duration of the experiments. In the mice treated with prolonged letrozole, administration of the drug was continued until resistance development. Resistance development was defined as the increase in the tumor volume of at least > 200% from the baseline tumor volume that sustained over a period of at least three consecutive weeks or more. Plasma from pooled cheek bleeds from five mice per treatment group was collected to measure Z-endoxifen concentrations as previously described [7]. As an exploratory extension of this experiment, *n* = 9 tumors that had developed letrozole resistance were randomized to Z-endoxifen (50 mg/kg) (*n* = 5) or tamoxifen (*n* = 4) for 4 weeks and tumors harvested for microarray and immunohistochemistry (IHC) analysis.

MCF7LR cells (2.2 × 10^6^ cells) in 100 μl of 1:1 phosphate-buffered saline (PBS) to Matrigel mixture were injected into the right flank of 6–7-week-old ovariectomized athymic nude female mice (Harlan Laboratories, Indianapolis, IN). Upon development of palpable tumors (tumor volume ≥ 150 mm^3^), the mice were randomized into five treatment groups (*n* = 12 mice/group) and were administered letrozole (10 μg/day) (control), fulvestrant (1000 μg/day), or exemestane (250 μg/day) (both drugs prepared as suspensions in 0.3% hydroxypropyl cellulose) subcutaneously, or Z-endoxifen (50 mg/kg), or everolimus (2.5 mg/day, prepared as suspensions in HPC:PEG400 50:50) by oral gavage 5 days a week. Mice in all treatment groups received the estrogen precursor androstenedione (100 μg/day), 5 days a week which is intratumorally converted by aromatase into estradiol [12]. Tumor volume and body weight were measured once a week as described above. When a group’s mean tumor volume reached ≥ 300% growth from the baseline volume, the mice in that group were euthanized and tumor tissue collected.

All animal studies were carried out according to the guidelines approved by the Institutional Animal Care and Use Committee (IACUC), at Mayo Clinic, Rochester, MN.

### Cell proliferation assay

Cells were seeded in 96-well plates (2000–4000 cells/well) and treated 24 h later with Z-endoxifen, tamoxifen, 4HT, fulvestrant, letrozole, or exemestane at the indicated concentrations. A week later, cells were fixed in situ with 25% (v/v) glutaraldehyde, followed by staining with 0.52% crystal violet (CV) in 25% methanol (Fisher Chemical). The CV stain was solubilized in 100 mM sodium citrate in 50% ethanol solution and absorbance of the stain measured at 550 nm using a plate reader. Each data point represents mean ± SD, obtained from six wells per treatment performed in biological triplicates. All reagents were from Sigma-Aldrich unless otherwise stated.

### Microarray analysis

MCF7LR tumors derived from letrozole treatment and following 4 weeks of randomized treatment to tamoxifen or Z-endoxifen were compared by Affymetrix Human Genome U133 Plus 2 Array to analyze the relative expression level of more than 47,000 transcripts and variants, including more than 38,500 well-characterized genes and unigenes by Mayo Clinic’s Advanced Genomics Technology Center, Rochester, MN. All analyses from the Affymetrix U133 Plus 2 Array were performed on the log base-2 scale. The perfect match gene expression probes were normalized and summarized using Tukey’s median polish to obtain one value per probe-set for each sample. Differential expression between pair-wise treatment groups were tested using Student’s *t* test. Genes were determined to be significantly regulated if their differential *P* value was ≤ 0.05. Fold changes were calculated by log 2 scale difference between the treatment and untreated groups. Validation of the results from the Affymetrix Array study was done by quantitative real-time polymerase chain reaction. The microarray data are deposited in NCBI’s Gene Expression Omnibus (GEO) repository, series accession number GSE146911.

Data were analyzed through the use of QIAGEN’s Ingenuity® Pathway Analysis (IPA®, www.qiagen.com/ingenuity). Briefly, the 532 regulated genes from the Z-endoxifen-treated groups (Additional file 7) and the 660 regulated genes from the tamoxifen-treated groups (Additional file 8) were used to perform IPA analysis. Statistically significant pathways in either Z-endoxifen- or tamoxifen-treated groups were first calculated using Fisher’s exact test (*p* value ≤ 0.05), followed by the Benjamini-Hochberg correction for multiple comparisons. We also conducted pathway analysis using GSEA and GSVA analysis to show the enrichment of ER signaling pathway in Tamoxifen compared to the Z-endoxifen. The enrichment score, *p* value, and false discovery rate (FDR) values have been obtained from the software.

### Quantitative real-time polymerase chain reaction

Total RNA from tissue samples were extracted using RNeasy Plus Mini Kit (Qiagen), and cDNA was generated using the ISCRIPT cDNA synthesis kit (Bio-Rad) using the manufacturer’s instructions. The qRT-PCR parameters used in this study are as follows: 1 cycle of 95 °C for 30 s followed by 40 cycles of 95 °C for 3 s and 60 °C for 30 s. Hypoxanthine-guanine phosphoribosyltransferase (*HPRT*) and Tubulin Alpha 1a (*TUBA1A*) were used as the endogenous reference genes. The primer sequences used were as follows: Amphiregulin (*AREG*) forward, 5′-ACT CGG CTC AGG CCA TTA TG-3′, and reverse, 5′-CGC TTC CCA GAG TAG GTG TCA-3′; Progesterone (*PGR*) forward, 5′-AAT GAA AGC CAA GCC CTA AGC-3′, and reverse, 5′-AAC AGG TTG ATC AGT GGT GGA A-3′; Trefoil factor 1 (*TFF1*) forward, 5′-CAC CAT GGA GAA CAA GGT GA-3′, and reverse, 5′-TGA CAC CAG GAA AAC CAC AA-3′; *HPRT* forward, 5′-CGT CTT GCT CGA GAT GTG ATG-3′, and reverse, 5′-GAG CAC ACA GAG GGC TAC AAT G-3′; *TUBA1A* forward, 5′-GAG TGC ATC TCC ATC CAC GTT-3′, and reverse, 5′-TAG AGC TCC CAG CAG GCA TT-3′. Target genes were normalized to the reference genes. Relative gene expression levels using the test and reference genes were calculated by the comparative Cq method.

### Western blot analysis

Serum-starved MCF7LR cells were treated with 1 μM letrozole, 1 nM androsteinedione, 0.1 μM or 5 μM concentrations of Z-endoxifen, tamoxifen, or 4HT for 1 h, and protein lysates were assessed for Akt (CS#9272), p-Akt (CS#9271), and Actin (CS#8457) (Cell Signaling, Danvers, MA) using 1:1000 antibody dilutions.

### Tissue processing and immunohistochemistry

Tumor tissues collected from Z-endoxifen and tamoxifen-randomized and letrozole-treated MCF7LR tumors were fixed overnight in buffered formalin (Fisher Scientific) and processed in the tissue core facility at Mayo Clinic (Scottsdale, AZ). Deparaffinized and rehydrated 5- to 6-μm sections were unmasked for 15 min in Citrate Buffer (pH 6.0) at 95 to 99 °C. Primary antibodies against phospho-Akt (Ser473) (CS#4060) at 1:100 dilution were incubated overnight at 4 °C. Secondary antibody (CS #8114) was applied for 30 to 60 min at room temperature. For Ki-67 staining of the tumoral tissues, primary antibodies against Ki-67 (Clone MIB-1) (Dako North America) at 1:600 were incubated overnight at 4 °C. Secondary antibody (Cell Signaling; SignalStain Boost IHC detection system #8125S) was applied for 30 to 60 min at room temperature. Chromogenic detection of protein expression was determined in the presence of 3,3′-diaminobenzidine (DAB) (BioCare) and visualized by light microscopy. Ki-67 was quantitated as percentage of tumor nuclei with staining.

### Statistical analysis

The primary outcome for comparing treatment groups was tumor volume, measured weekly using calipers and calculated as (width^2^ × length)/2. Longitudinal measures of tumor volume were used to create tumor volume growth curves. Then, the area under the curve (AUC) was calculated as a summary measure for tumor volume change (growth or shrinkage relative to baseline) for each mouse; the AUC was estimated using the trapezoid method. AUC values were compared between groups using Wilcoxon rank-sum tests unless otherwise stated. For descriptive purposes, the mean and the standard error of the mean (SEM) of the longitudinal tumor volumes as a percent of baseline (i.e., 100 × (follow-up tumor volume/baseline tumor volume)) were plotted over time for each group. Differences in mouse body weight between treatment groups were also compared using Wilcoxon rank-sum tests at specified time points.

For the extension of the first experiment, in which letrozole-treated MCF7AC1 tumors were followed until they developed letrozole resistance (MCF7LR) and then were either selected for randomization to Z-endoxifen or tamoxifen or continued on letrozole, two-sample *t* tests were used to compare groups because of the very small sample size (3–5 mice per group) and limited power for detecting differences; the outcomes compared in this exploratory extension of the first experiment included Ki67 nuclear expression and tumor volume AUC during 4 weeks of treatment. Analysis was performed using SAS (version 9.4). Differences in the mRNA expression of *AREG*, *PGR*, and *TFF1* genes between SERM-treated MCF7LR tumors relative to letrozole-treated MCF7LR tumors that were normalized to 1.0 were analyzed by one sample *t* test using imaging software Graphpad Prism (version 8.0.2). A *P* value of < 0.05 was considered statistically significant.

## Results

### The effect of Z-endoxifen on the growth of AI-sensitive and AI-resistant ER+ breast cancer in vivo

**Fig. 1:**
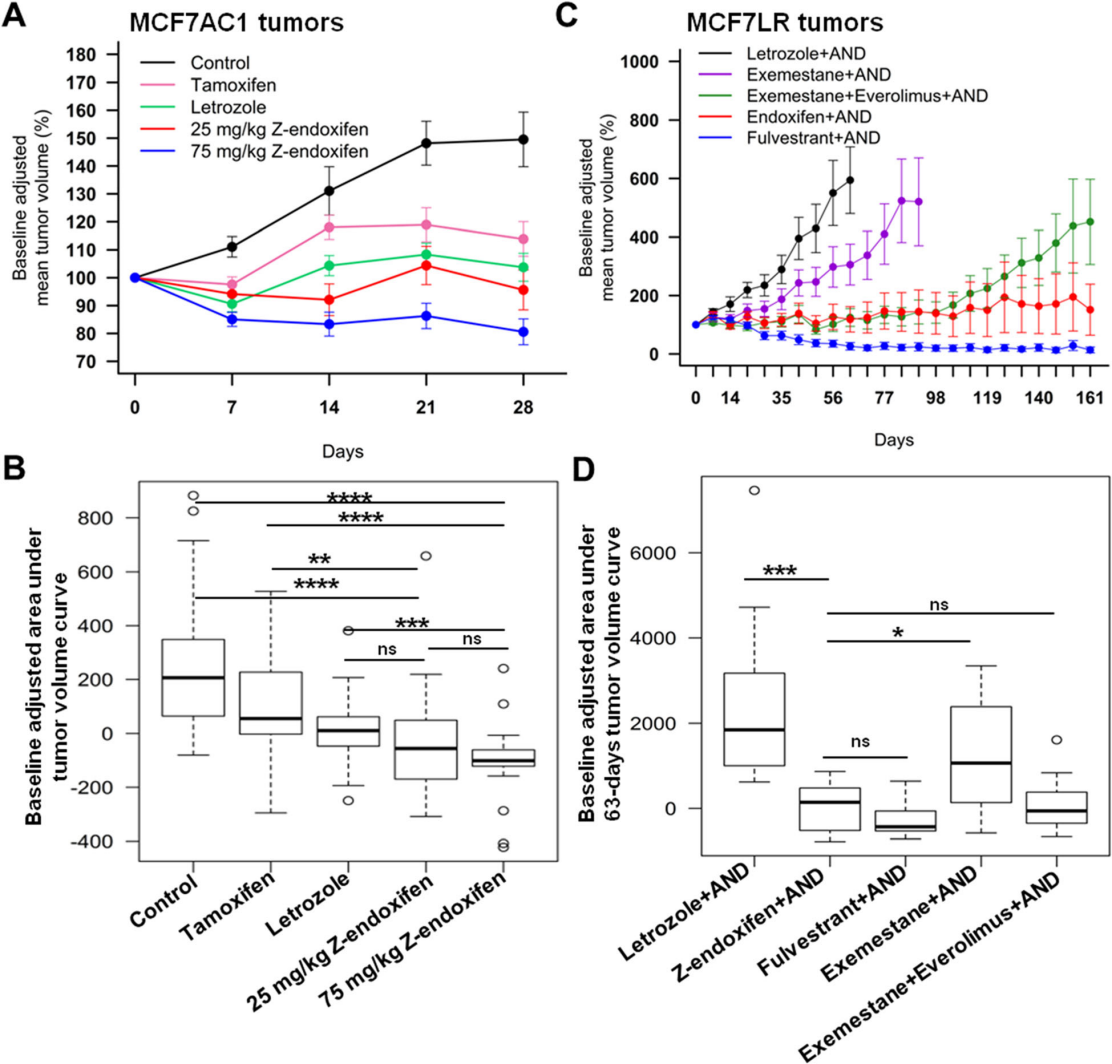
Z-endoxifen and standard endocrine therapies efficacy on AI-sensitive and AI-resistant tumors growth in vivo. **a** Four- to 6-week-old female ovariectomized BALB/c athymic female nude mice were subcutaneously injected with 2.5 × 10^6^ MCF7AC1 cells in 100 μl of 1:1 phosphate-buffered saline (PBS) to Matrigel mixture in the right and left flanks. When tumor volumes reached ≥ 300 mm^3^, mice were randomized (*n* = 30 mice/group) to control, letrozole (10 μg/day), tamoxifen (500 μg/day), or Z-endoxifen (25 mg/kg and 75 mg/kg) treatment. Tumor volume was assessed every week for a period of 4 weeks. Data are presented as mean ± SEM. **b** The area under the tumor volume growth curve (AUC), adjusted for baseline, was calculated through 4 weeks of treatment; AUC distributions by treatment group are shown with side-by-side boxplots and were compared between groups using Wilcoxon rank-sum tests. **c** Six- to seven-week-old female nude mice were injected with 2.2 × 10^6^ MCF7LR cells in the right flank. When tumor volumes reached ≥ 150 mm^3^, mice were randomized (*n* = 12 mice/group) to letrozole, Z-endoxifen (50 mg/kg), exemestane (250 μg/day) alone, or exemestane plus everolimus (2.5 mg/day). Tumor volume was measured every week. Data are presented as mean ± SEM. **d** The area under the tumor volume growth curve (AUC), adjusted for baseline, was calculated through 63 days (9 weeks) of treatment; AUC distributions by treatment group are shown with side-by-side boxplots and were compared between groups using Wilcoxon rank-sum tests. Non-significant (ns), *P* > 0.05; *P < 0.05; **P < 0.01; ***P < 0.001

Assessment of the tumor volume at 4 weeks in the AI-sensitive MCF7AC1 xenografted mice treated with control (*n* = 28), letrozole (*n* = 29), tamoxifen (*n* = 30), or Z-endoxifen [25 mg/kg (*n* = 27) or 75 mg/kg (*n* = 26)] revealed that the 75 mg/kg Z-endoxifen treatment was superior in reducing tumor volume, analyzed using the area under the curve, compared to control (*p* < 0.0001), tamoxifen (*p* < 0.0001), and letrozole (*p* = 0.0005) treatments while the 25 mg/kg Z-endoxifen treatment was superior to control (*p* < 0.0001) and tamoxifen (*p* = 0.002) with a trend towards greater benefit compared to letrozole (*p* = 0.10) (Fig. 1a and b). Treatment with 75 mg/kg Z-endoxifen significantly reduced murine body weight at 4 weeks (Additional file 1). In order to assess the bioavailability of Z-endoxifen, plasma concentrations of Z-endoxifen were analyzed in mice treated with tamoxifen and Z-endoxifen (25 mg/kg and 75 mg/kg) at 2 weeks. Direct oral administration of 25 and 75 mg/kg Z-endoxifen yielded plasma concentrations of Z-endoxifen of 11.8 ng/ml and 391.3 ng/ml at 2 weeks. In contrast, Z-endoxifen concentrations were nearly undetectable (0 ng/ml) in tamoxifen-treated group.

In an effort to develop a letrozole-resistant tumor model, we extended treatment in the letrozole arm and letrozole resistance emerged in a subset (9 out of 17 mice that survived prolonged treatment) beginning at 25 weeks (Additional file 2a). At 27 weeks, letrozole treatment was discontinued and the subset harboring letrozole-resistant tumors (MCF7LR) (*n* = 9) were randomized to either Z-endoxifen (oral, 50 mg/kg) (*n* = 5) or tamoxifen (subcutaneous, 500 μg/day) (*n* = 4) for 4 weeks. Owing to weight loss issues previously observed with 75 mg/kg Z-endoxifen at 4 weeks, a reduced dose of 50 mg/kg Z-endoxifen was chosen for the extended experiment. In these MCF7LR tumors, 50 mg/kg Z-endoxifen significantly reduced tumor volume compared to tamoxifen (*p* = 0.045) (Additional file 2b).

Given the promising antitumor activity of Z-endoxifen, we initiated a second in vivo study wherein the antitumor activity of Z-endoxifen was compared with additional endocrine regimens with known efficacy in AI-resistant breast cancer. MCF7LR tumor-bearing mice were treated with letrozole, Z-endoxifen (50 mg/kg), fulvestrant, exemestane, or exemestane plus everolimus (an mTOR inhibitor) and their effects on tumor growth assessed in vivo. Treatment with letrozole, to which the tumors acquired resistance, failed to inhibit growth with the mean tumor volume reaching > 200% from baseline treatment at 10 weeks, whereas treatment with fulvestrant, a known potent selective estrogen receptor degrader (SERD), markedly inhibited tumor growth (Fig. 1c). Additionally, tumors treated with exemestane alone as well as exemestane plus everolimus were growth delayed compared to the letrozole alone, with the mean tumor volume reaching > 200% from baseline treatment in the exemestane arm at 13 weeks and in the exemestane plus everolimus arm at 23 weeks (Fig. 1c). Throughout this period, Z-endoxifen potently inhibited tumor growth with reduced mean tumor volume and its antitumor activity, quantified as the area under the longitudinal tumor volume curve through 9 weeks (63 days) of treatment (at which point mice in the letrozole treatment group were sacrificed), was superior to letrozole (*p* < 0.001) and exemestane (*p* = 0.03) (Fig. 1c and d) but not significantly different from exemestane plus everolimus (*p* = 0.93) or fulvestrant (*p* = 0.29). As previously observed, body weight was significantly reduced in the Z-endoxifen-treated mice compared to the other treatments (Additional file 3). However, addition of oral nutritional supplement Nutrical (Webster Veterinary) in the diet of mice receiving Z-endoxifen treatment helped to control the weight loss issues in these mice, allowing us to continue with the Z-endoxifen treatment.

**Fig. 2:**
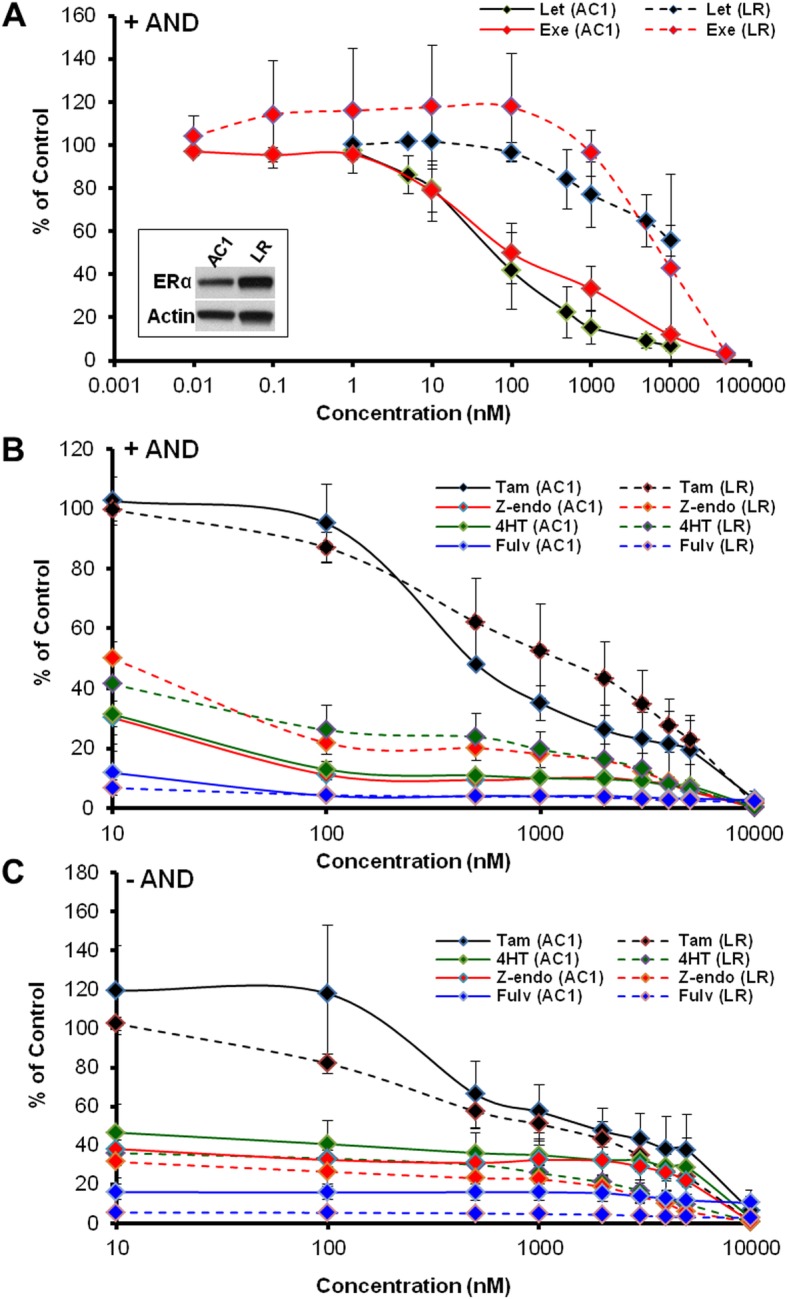
The effects of AIs, SERMs and fulvestrant on MCF7AC1 and MCF7LR cell growth in vitro. **a** Treatment with letrozole and exemestane in the presence of AND for 7 days. **b** Treatment with tamoxifen, Z-endoxifen, 4HT, and fulvestrant in the presence of AND for 7 days. **c** Treatments with aforementioned drugs mentioned in (**b**) in the absence of AND for 7 days. Growth was assessed by fixing the cells in 25% (v/v) glutaraldehyde followed by staining with 0.52% crystal violet in 25% methanol. Data is representative of six wells per treatment performed in biological triplicates and presented as mean ± SD. AND, androstenedione

With the emergence of the MCF7LR tumors, these tumors were harvested from in vivo and the letrozole-resistant MCF7LR cell line was established in vitro. Next, we characterized Z-endoxifen effects on the growth of AI-sensitive and AI-resistant breast cancer cell lines in vitro. First, we confirmed that the AI-resistant MCF7LR cells retained resistance to AI treatments (letrozole and exemestane) in vitro (Fig. 2a). Evaluation of estrogen receptor α (ERα) expression, the target of Z-endoxifen, revealed increased ERα expression in MCF7LR cells compared to MCF7AC1 cells (Fig. 2a, inset). Consistent with the in vivo data, the anti-proliferative effects of Z-endoxifen in androstenedione-treated MCF7AC1 (sensitive) and MCF7LR (resistant) cells were superior to tamoxifen and similar to fulvestrant. Evaluation of 4HT mirrored the antitumor effects of Z-endoxifen (Fig. 2b). These drugs produced the same effects even when these cells were grown in the absence of androstenedione (Fig. 2c).

To determine whether Z-endoxifen actions in ER+ breast cancer cells were affected by HER2 status, we also evaluated Z-endoxifen in cell lines that do not express HER2 (T47D), endogenously express HER2 (BT474), or stably over-express HER2 (MCF7-HER2–18). We also confirmed ERα expression in these cell lines (Additional file 4e). Z-endoxifen exhibited superior antiproliferative effects over tamoxifen in inhibiting growth of these estrogen-dependent cells, while 4HT and fulvestrant effects were akin to the outcomes observed in MCF7AC1 and MCF7LR cells (Additional file 4a-d). Taken together, these data suggest that Z-endoxifen exhibits broad antitumor activity in ER+ breast cancer cells regardless of HER2 status. Lastly, we evaluated the antitumor activity of the Z-endoxifen, tamoxifen, and 4HT in ER− and HER2− breast cancer cells (MDAMB231, MDAMB468, and BT20). In these cell lines, Z-endoxifen exhibited antiproliferative activity at concentrations > 5 μM, with the pattern of antiproliferative activity similar to that of tamoxifen and 4HT, both in the presence and absence of estrogen (Additional file 5a and b).

### The transcriptome of Z-endoxifen- and tamoxifen-treated letrozole-resistant tumors

In the MCF7LR tumors harvested after 4 weeks of therapy with either tamoxifen or Z-endoxifen, IHC analyses for nuclear Ki67 expression was obtained and compared to letrozole-treated MCF7LR tumors not exposed to either Z-endoxifen or tamoxifen. Despite the fact that tumor volumes were significantly lower in the Z-endoxifen arm compared to the tamoxifen arm (Additional file 2), both Z-endoxifen (*p* = 0.0005) and tamoxifen (*p* = 0.008) significantly suppressed nuclear Ki-67 expression compared to letrozole-treated MCF7LR tumors (Additional file 6a and b).

**Fig. 3:**
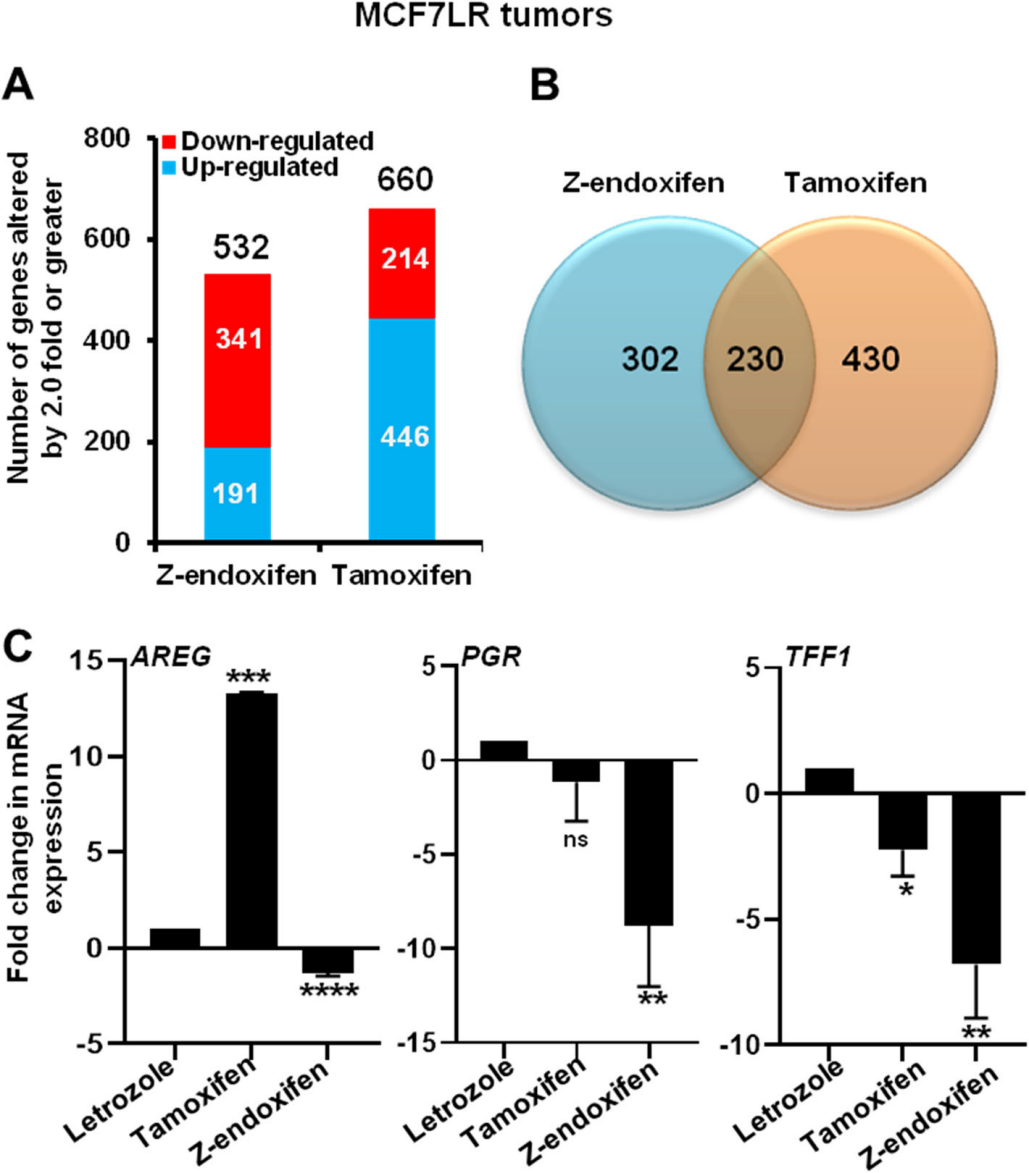
The effects of Z-endoxifen and tamoxifen treatment on gene expression in the MCF7LR tumors. **a** Graph depicting the number of genes significantly upregulated (blue) and downregulated (red) 2.0-fold or greater by the indicated SERM treatments in the MCF7LR tumors compared to letrozole-treated MCF7LR tumors. **b** Venn diagram of genes whose mRNA expression levels were significantly altered by 2.0-fold or greater in Z-endoxifen- or tamoxifen-treated MCF7LR tumors compared to letrozole-treated MCF7LR tumors. **c** Gene expression of *AREG*, *PGR*, and *TFF1* as analyzed by quantitative polymerase chain reaction (performed in triplicate wells per gene) in Z-endoxifen- or tamoxifen-treated MCF7LR tumors compared to letrozole-treated MCF7LR tumors. Data is representative of three wells per gene performed in biological duplicates and presented as mean ± SD. Difference in gene expression in the SERM-treated MCF7LR tumors compared to letrozole-treated tumors that are normalized to 1.0 were compared by one sample *t* test. *AREG,* Amphiregulin; *PGR*, progesterone; *TFF1*, Trefoil factor 1. ns, *P* > 0.05; *P < 0.05; **P < 0.01; ***P < 0.001 compared to letrozole treatment

Therefore, in order to explore the mechanisms by which Z-endoxifen exerted its antitumor activity and superiority over tamoxifen, we compared the transcriptome of each of these MCF7LR tumors with letrozole-treated MCF7LR tumors for 4 weeks to identify genes, and their associated biological pathways, that were differentially regulated by Z-endoxifen and tamoxifen. Z-endoxifen significantly regulated the expression of 532 genes of which 64% (341 genes) were downregulated and 36% (191 genes) were upregulated (Fig. 3a). Tamoxifen significantly regulated the expression of 660 genes, of which 32% (214 genes) were downregulated and 68% (446 genes) were upregulated (Fig. 3a). Two hundred thirty genes were commonly regulated by both Z-endoxifen and tamoxifen treatments, of which 223 genes were concordant and 7 genes were discordant between the two SERMs (Fig. 3b, Additional file 9). We focused on genes whose expression was differentially regulated as they were more likely to contribute to the opposite in vivo growth phenotypes observed between the two SERM treatment groups*.* Of the seven discordantly regulated genes, the mRNA expression of *AREG*, an estrogen-regulated gene [13], was significantly inhibited by Z-endoxifen (− 3.2 fold, *p* = 0.0006). Conversely, tamoxifen significantly induced *AREG* mRNA expression (+ 9.2 fold, *p* = 0.00002) (Additional files 7 and 8). *PGR*, another estrogen-regulated gene [14], was significantly downregulated by Z-endoxifen (− 4.8 fold, *p* = 4.34 × 10^−8^) but was unchanged with tamoxifen treatment (Additional files 7 and 8). qRT-PCR analysis validated these findings (Fig. 3c). Further evaluation of another estrogen-regulated gene, *TFF1* [15], by qRT-PCR analysis showed that Z-endoxifen suppressed *TFF1* mRNA expression (− 6.8 fold, *p* = 0.0054) more profoundly than tamoxifen (− 2.2 fold, *p* = 0.032) in the MCF7LR tumors (Fig. 3c). Ingenuity pathway analysis (IPA) performed to identify canonical pathways significantly impacted by Z-endoxifen and tamoxifen in the MCF7LR tumors revealed estrogen-mediated S-phase entry pathway, as one of the top ten pathways significantly impacted by Z-endoxifen (*p* = 0.0029). Interestingly, in the tamoxifen-treated resistant tumors, none of the top ten pathways altered by tamoxifen were estrogen-dependent (Additional files 10 and 11). IPA analysis further identified ATM signaling (*p* = 0.0037) and PI3K/AKT signaling (*p* = 0.0129) pathways to be significantly and uniquely inhibited by Z-endoxifen but not by tamoxifen (Additional files 10 and 11). Consistent with the IPA study, gene set enrichment analysis (GSEA) [16] indicated that the estrogen signaling pathway was significantly enriched in Z-endoxifen (*p* < 0.001) but not in tamoxifen-treated (*p* = 0.151) MCF7LR tumors (Additional file 12). Additionally, gene set variation analysis (GSVA), which provides increased power to detect subtle pathway activity changes over a sample population [17], further validated the outcomes of the GSEA analysis (Additional file 13).

### Effects of Z-endoxifen on kinase-associated signaling pathways

**Fig. 4:**
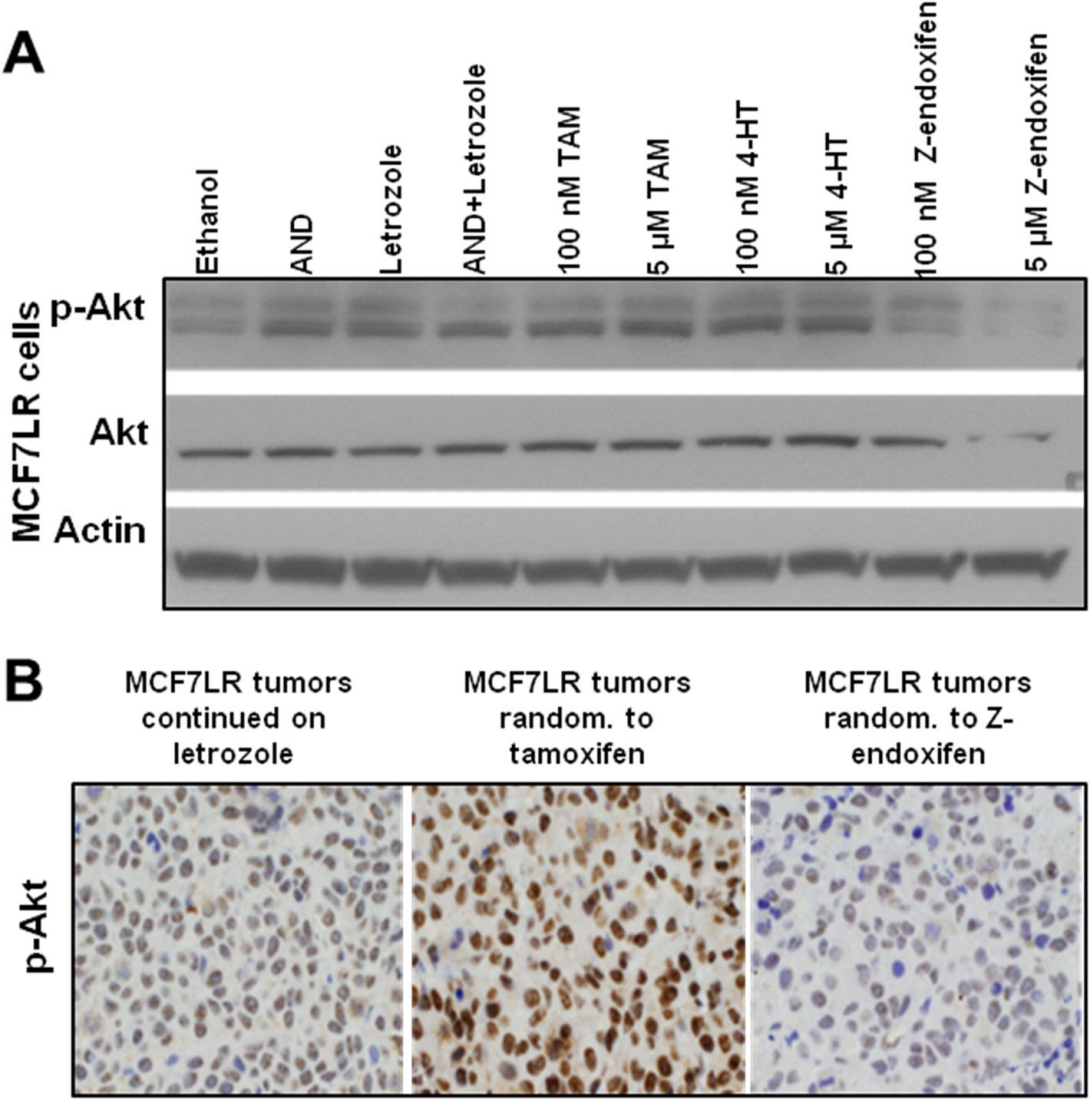
The effect of Z-endoxifen on the protein expression of Akt in the setting of letrozole resistance. **a** Treatment of MCF7LR cells with the indicated treatments for 1 h followed by immunoblotting for detection of phospho and total Akt and the loading control Actin. Images are representative of two independent experiments. **b** IHC staining of Z-endoxifen-, tamoxifen- (*n* = 3), and letrozole-treated MCF7LR tumors for p-Akt protein. Images are representative of at least three independent tissue staining. AND, androsteinedione; TAM, tamoxifen

Based on the IPA findings, we evaluated Z-endoxifen effects on Akt kinase protein expression. First, we evaluated the effects of letrozole (1 μM) and low (100 nM) or high (5 μM) concentrations of Z-endoxifen, tamoxifen, or 4HT in MCF7LR cells following 1 h of treatment. Z-endoxifen concentration of 5 μM effectively inhibited phospho and total Akt protein levels relative to ethanol (control), whereas letrozole, tamoxifen, and 4HT treatments induced p-Akt levels (Fig. 4a). Consistent with this in vitro observation, Z-endoxifen reduced while tamoxifen induced p-Akt levels in the MCF7LR tumors compared to MCF7LR tumors receiving letrozole treatment, as revealed by IHC analysis (Fig. 4b).

## Discussion

The MCF7AC1 cell line has proven to be an ideal model system for evaluating the in vivo effects of AIs and antiestrogenic therapies. In prior studies, this preclinical model not only established the superiority of letrozole (AI) over tamoxifen in the first-line setting but also demonstrated efficacy of second-line letrozole therapy in the treatment of tumors that had progressed on tamoxifen [10]. Utilizing this same model, in the MCF7AC1 tumors, we have shown that both the 25 mg/kg and 75 mg/kg doses of Z-endoxifen display superior antitumor activity compared to control and tamoxifen. Additionally, in the letrozole-resistant MCF7LR model, we have shown that Z-endoxifen has superior efficacy to tamoxifen and exemestane monotherapy and was similar to exemestane and everolimus, regimens which have demonstrated proven efficacy in patients with resistance to non-steroidal AIs. Given that treatments with letrozole, exemestane, and exemestane plus everolimus in the MCF7LR tumors resulted in tumor resistance occurring before or by 23 weeks, it is worth mentioning that Z-endoxifen administration even as far as out to 23 weeks remarkably did not result in tumor resistance in vivo, indicating that prolonged Z-endoxifen treatment may not result in cross resistance. Because the 75 mg/kg dose of Z-endoxifen significantly reduced body weight, we evaluated the 50 mg/kg dose in the letrozole-resistant MCF7LR model and showed substantial antitumor activity compared to tamoxifen. In a subsequent in vivo experiment, we also demonstrated greater efficacy compared to letrozole and exemestane as well as a trend towards superiority compared to exemestane plus everolimus. Although, we do not know whether the 50 mg/kg would be superior to letrozole in the letrozole-sensitive MCF7AC1 tumors, no significant differences were observed in the antitumor activity comparing the 25 mg/kg and 75 mg/kg doses of Z-endoxifen. Previous studies have reported that 50 mg/kg endoxifen administration into ovariectomized C57BL/6 mice bearing no xenograft tumors significantly reduced body weight compared to nontreated mice [18]. Based on this observation, it appears the reduced body weight noted in our study in Z-endoxifen-treated mice may likely be a non-tumor effect and calls for further investigation of the potential link between Z-endoxifen and weight loss.

Limited pK studies with the Z-endoxifen 75 mg/kg dose at 2 weeks post drug treatment demonstrated high circulating drug concentrations. Of note, Z-endoxifen administered orally at a dose of 80 mg/day in humans resulted in plasma concentrations of 1–2 μM Z-endoxifen [9], which appears to be comparable to the concentration of Z-endoxifen achieved in mice with the 75 mg/kg dose at 2 weeks (1.05 μM) in this study. Our data also reinforce observations from previous studies that direct oral administration of Z-endoxifen results in substantially higher Z-endoxifen plasma concentrations compared to the Z-endoxifen levels achieved via tamoxifen administration [7].

The inability of tamoxifen to curb tumor growth in the MCF7LR tumors could possibly be explained by the observation that tamoxifen predominantly induced gene expression (68%) in the resistant tumors, whereas Z-endoxifen on the contrary predominantly suppressed gene expression (64%) in these tumors. Moreover, IPA studies also revealed stark discordance in the biological pathways impacted by Z-endoxifen and tamoxifen, lending further support to the observation that the mode of action of Z-endoxifen is distinct from that of its parent drug tamoxifen. Supporting this notion, the growth-promoting estrogen-mediated ERα signaling [19] and its downstream target genes (*AREG*, *PGR*, and *TFF1*) were significantly downregulated only in the Z-endoxifen-treated, but not in the tamoxifen-treated, MCF7LR tumors, supporting previous observations that Z-endoxifen is a more potent antiestrogen than tamoxifen [3].

ERα is considered the major target of Z-endoxifen with studies showing altered ERα expression in Z-endoxifen-treated cells [3]. However, both immediate in vitro (1 h) and long-term in vivo reduction in the protein expression of Akt kinase, which is implicated in tumor progression and drug resistance [20, 21], in Z-endoxifen-treated MCF7LR tumors, suggest that Z-endoxifen may have additional roles beyond targeting ERα in ER+ breast cancer. Therefore, further studies should focus on identifying signaling kinases that are targeted by Z-endoxifen in order to better understand the non-estrogenic mechanisms through which Z-endoxifen imparts its antitumor activity. This knowledge will help clinicians to identify patient cohorts who might benefit from Z-endoxifen treatment, based on the expression status of these kinases in the tumor tissues.

In summary, we have shown that in AI-sensitive and AI-resistant breast tumors, Z-endoxifen display superior antitumor and antiestrogenic activity compared to tamoxifen. The observation of substantial Z-endoxifen antitumor activity in letrozole-resistant tumors supports the ongoing clinical studies in AI refractory breast cancer. Given the marked differences in Akt expression, further studies are warranted to assess the effects of Z-endoxifen on additional signaling kinases.

## Conclusion

In summary, the present study demonstrates that in the AI-sensitive and AI-resistant tumors, Z-endoxifen results in robust antitumor and antiestrogenic activity compared to tamoxifen and AI monotherapy. Importantly, in the AI-resistant MCF7LR tumors, prolonged Z-endoxifen therapy did not result in cross resistance when compared to endocrine therapies with known efficacy in AI-resistant breast cancer including letrozole and exemestane monotherapy and extemestane plus everolimus combinatorial therapy. These findings indicate that Z-endoxifen may provide clinical benefit over AI’s and tamoxifen in AI-resistant breast tumors lending further support to the ongoing development of Z-endoxifen.

## Supplementary information

**Additional file 1. **The effect of Z-endoxifen on the body weight of MCF7AC1 tumors harboring mice. The graph represents the average body weight of the mice in the control (*n* = 28), tamoxifen (*n* = 30), letrozole (*n* = 29), 25 mg/kg (*n* = 27) and 75 mg/kg (*n* = 26) Z-endoxifen treatment groups measured at four weeks. Data are presented as mean ± SD. Differences in the body weight between the treatments were compared using Wilcoxon rank-sum tests. ***, *P* < 0.001; ****, *P* < 0.0001 compared to 75 mg/kg Z-endoxifen treatment group.

**Additional file 2. **Development of the letrozole-resistant MCF7LR tumors in vivo. a For the development of letrozole-resistant tumors, xenografted MCF7AC1 tumors were chronically exposed to letrozole therapy. At 25 weeks, a subset of tumor-bearing mice in the letrozole group (*n* = 9) developed resistance to letrozole therapy in vivo. At 27 weeks, letrozole-resistant (MCF7LR) mice were randomized to tamoxifen (*n* = 4) or Z-endoxifen (*n* = 5) treatments. Data are presented as mean ± SEM. b Dotplot displaying the baseline-adjusted area under the tumor volume curve for the MCF7LR tumor-bearing mice randomized to Z-endoxifen or tamoxifen treatments. The *p*-value was calculated by two-sample t-test. *, *P* < 0.05.

**Additional file 3. **The effect of Z-endoxifen on the body weight of MCF7LR tumors harboring mice. The graph represents the average body weight of the mice (*n* = 12/group) in the each treatment group measured at 63 days. Data are presented as mean ± SD. Differences in the body weight of mice between the treatments over the treatment duration were compared using Wilcoxon rank-sum tests. *, *P* < 0.05; **, *P* < 0.01 compared to Z-endoxifen+AND treatment group.

**Additional file 4. **The effects of the SERMs and fulvestrant on the growth of T47D, BT474, neo and HER2/18-expressing MCF7 cells in vitro. **a-d** Treatment of the cells with tamoxifen, Z-endoxifen, 4HT and fulvestrant in the presence of 1 nM E2 at the indicated concentrations for seven days. Growth was assessed by fixing the cells in glutaraldehyde followed by staining with crystal violet. Data is representative of six wells per treatment performed in biological triplicates and presented as mean ± SD. **e** Western blot analysis of HER2 and ERα expression in the indicated cell lines. Actin served as the loading control. Images are representative of three independent experiments. E2 = Estradiol.

**Additional file 5. **The effects of the SERMs on the growth of MDAMB231, MDAMB468 and BT20 cells in vitro. **a** Treatment of the cells with tamoxifen, Z-endoxifen and 4HT in the absence of estradiol for seven days. **b** Treatment with the aforementioned drugs in the presence of 1 nM E2 for seven days. Growth was assessed by fixing the cells in glutaraldehyde followed by staining with crystal violet. Data is representative of six wells per treatment performed in biological triplicates and presented as mean ± SD. E2 = Estradiol.

**Additional file 6. **The effect of Z-endoxifen and tamoxifen on Ki67 protein in MCF7LR tumors. **a** Ki67 expression in letrozole (*n* = 3), Z-endoxifen (*n* = 5) or tamoxifen (n = 3) treated MCF7LR tumors analyzed by IHC. **b** Histogram of the percentage of Ki67 nuclear staining in these tumors. Differences in the gene expression in the SERM-treated MCF7LR tumors compared to the letrozole-treated MCF7LR tumors were compared using two-sample t-tests. Non-significant (ns), *P* > 0.05; **, *P* < 0.01; ***, *P* < 0.001 compared to Continued Letrozole treatment group.

**Additional file 7.** List of 532 genes regulated by Z-endoxifen treatment in MCF7LR tumors compared to letrozole-treated MCF7LR tumors.

**Additional file 8.** List of 660 genes regulated by tamoxifen treatment in the MCF7LR tumors compared to letrozole-treated MCF7LR tumors.

**Additional file 9.** List of genes commonly regulated by Z-endoxifen or tamoxifen treatments in the MCF7LR tumors compared to letrozole-treated MCF7LR tumors.

**Additional file 10.** Ingenuity pathway analysis (IPA) indicating key molecular, cellular and signaling pathways likely impacted by Z-endoxifen treatment in the MCF7LR tumors.

**Additional file 11.** Ingenuity pathway analysis (IPA) indicating key molecular, cellular and signaling pathways likely impacted by tamoxifen treatment in the MCF7LR tumors.

**Additional file 12 **Gene Set Enrichment Analysis (GSEA) of the estrogen signaling pathway. **a** The pathway enrichment score of the estrogene signaling pathway (from Kyoto Encyclopedia of Genes and Genome (KEGG)) in the treatment groups along with the nominal *p*-values and false discovery rate (FDR) q-values. **b** Enrichment plot of the KEGG estrogen signaling pathway of Z-endoxifen-treated MCF7LR tumors compared with letrozole-resistant MCF7LR tumors. The enrichment score and the *p*-value are listed.

**Additional file 13 **Summary of Gene Set Variation Analysis (GSVA) for the estrogen signaling pathway. **a** The Table shows the enrichment score values of the estrogen signaling pathway for individual samples and the treatment groups. **b** The GSVA enrichment scores for the estrogen signaling pathway between the treatment groups. **c** Table shows the group mean value and *p*-value of the different comparisons.

## Data Availability

The datasets used and/or analyzed in this study are available from the corresponding author on reasonable request.

## Abbreviations

ER: Estrogen receptor
SERM: Selective estrogen receptor modulator
4HT: 4-Hydroxytamoxifen
CYP2D6: Cytochrome P450 2D6
AI: Aromatase inhibitor
HER2: Human epidermal growth factor receptor 2
PBS: Phosphate-buffered saline
AREG: Amphiregulin
PGR: Progesterone
TFF1: Trefoil factor 1
HPRT: Hypoxanthine-guanine phosphoribosyltransferase
TUBA1A: Tubulin Alpha 1a
GEO: Gene Expression Omnibus
FDR: False discovery rate
KEGG: Kyoto Encyclopedia of Genes and Genome
GSEA: Gene set enrichment analysis
GSVA: Gene variation enrichment analysis
