# Supplementary material for: Antitumor activity of Z-endoxifen in aromatase inhibitor-sensitive and aromatase inhibitor-resistant estrogen receptor-positive breast cancer
Source: Breast Cancer Res. 2020 May 19;22:51. doi: 10.1186/s13058-020-01286-7 (PMC7238733; doi:10.1186/s13058-020-01286-7)
Supplement: Supplementary file 1 — Additional file 1. The effect of Z-endoxifen on the body weight of MCF7AC1 tumors harboring mice. The graph represents the average body weight of the mice in the control (n = 28), tamoxifen (n = 30), letrozole (n = 29), 25 mg/kg (n = 27) and 75 mg/kg (n = 26) Z-endoxifen treatment groups measured at four weeks. Data are presented as mean ± SD. Differences in the body weight between the treatments were compared using Wilcoxon rank-sum tests. ***, P < 0.001; ****, P < 0.0001 compared to 75 mg/kg Z-endoxifen treatment group. [file 13058_2020_1286_MOESM1_ESM.docx]

**Additional file 1:**

**
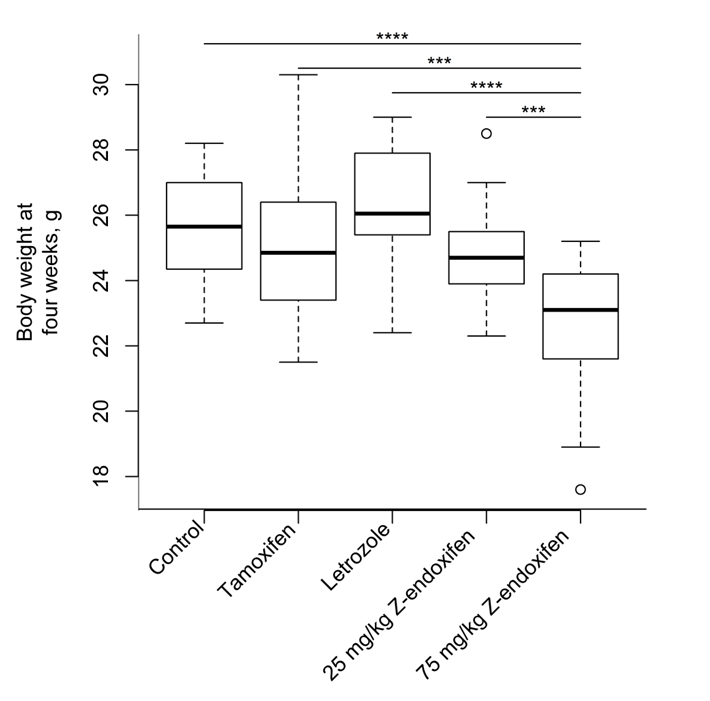
**

**Figure S1. The effect of Z-endoxifen on the body weight of MCF7AC1 tumors harboring mice.** The graph represents the average body weight of the mice in the control (n=28), tamoxifen (n=30), letrozole (n=29), 25 mg/kg (n=27) and 75mg/kg (n=26) Z-endoxifen treatment groups measured at four weeks. Data are presented as mean ± SD. Differences in the body weight between the treatments were compared using Wilcoxon rank-sum tests. ***, *P* < 0.001; ****, *P* < 0.0001 compared to 75 mg/kg Z-endoxifen treatment group.
