# Supplementary material for: Antitumor activity of Z-endoxifen in aromatase inhibitor-sensitive and aromatase inhibitor-resistant estrogen receptor-positive breast cancer
Source: Breast Cancer Res. 2020 May 19;22:51. doi: 10.1186/s13058-020-01286-7 (PMC7238733; doi:10.1186/s13058-020-01286-7)
Supplement: Supplementary file 2 — Additional file 2. Development of the letrozole-resistant MCF7LR tumors in vivo. a For the development of letrozole-resistant tumors, xenografted MCF7AC1 tumors were chronically exposed to letrozole therapy. At 25 weeks, a subset of tumor-bearing mice in the letrozole group (n = 9) developed resistance to letrozole therapy in vivo. At 27 weeks, letrozole-resistant (MCF7LR) mice were randomized to tamoxifen (n = 4) or Z-endoxifen (n = 5) treatments. Data are presented as mean ± SEM. b Dotplot displaying the baseline-adjusted area under the tumor volume curve for the MCF7LR tumor-bearing mice randomized to Z-endoxifen or tamoxifen treatments. The p-value was calculated by two-sample t-test. *, P < 0.05. [file 13058_2020_1286_MOESM2_ESM.docx]

**
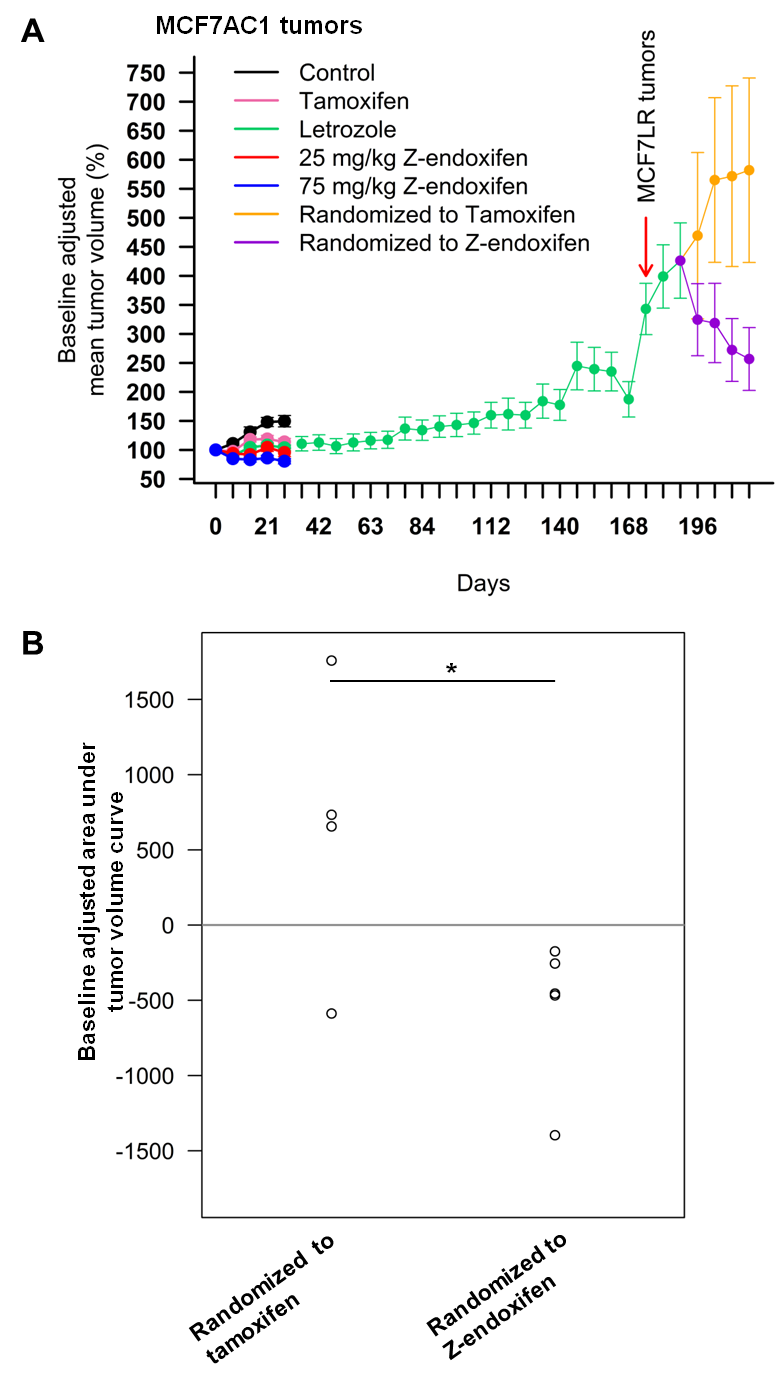
Additional file 2:**

**Figure S2. Development of the letrozole-resistant MCF7LR tumors *in vivo*. a** For the development of letrozole-resistant tumors, xenografted MCF7AC1 tumors were chronically exposed to letrozole therapy. At 25 weeks, a subset of tumor-bearing mice in the letrozole group (n=9) developed resistance to letrozole therapy *in vivo*. At 27 weeks, letrozole-resistant (MCF7LR) mice were randomized to tamoxifen (n=4) or Z-endoxifen (n=5) treatments. **b** Dotplot displaying the baseline-adjusted area under the tumor volume curve for the MCF7LR tumor-bearing mice randomized to Z-endoxifen or tamoxifen treatments. The p-value was calculated by two-sample t-test. *, *P* < 0.05.
