# Supplementary material for: Antitumor activity of Z-endoxifen in aromatase inhibitor-sensitive and aromatase inhibitor-resistant estrogen receptor-positive breast cancer
Source: Breast Cancer Res. 2020 May 19;22:51. doi: 10.1186/s13058-020-01286-7 (PMC7238733; doi:10.1186/s13058-020-01286-7)
Supplement: Supplementary file 3 — Additional file 3. The effect of Z-endoxifen on the body weight of MCF7LR tumors harboring mice. The graph represents the average body weight of the mice (n = 12/group) in the each treatment group measured at 63 days. Data are presented as mean ± SD. Differences in the body weight of mice between the treatments over the treatment duration were compared using Wilcoxon rank-sum tests. *, P < 0.05; **, P < 0.01 compared to Z-endoxifen+AND treatment group. [file 13058_2020_1286_MOESM3_ESM.docx]

**Additional file 3:**

**
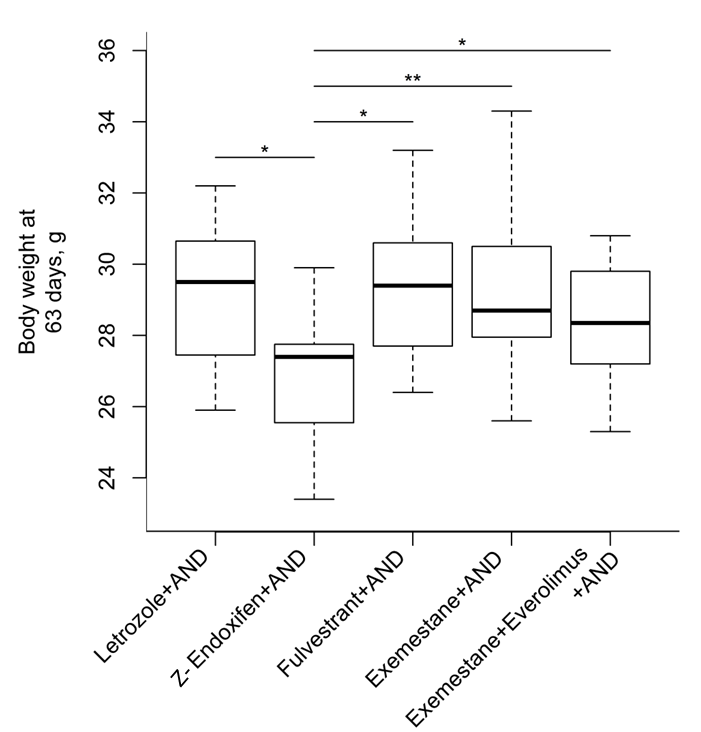
**

**Figure S3. The effect of Z-endoxifen on the body weight of MCF7LR tumors harboring mice.** The graph represents the average body weight of the mice (n=12/group) in the each treatment group measured at 63 days. Data are presented as mean ± SD. Differences in the body weight of mice between the treatments over the treatment duration were compared using Wilcoxon rank-sum tests. *, *P* < 0.05; **, *P* < 0.01 compared to Z-endoxifen+AND treatment group.
