# Supplementary material for: Antitumor activity of Z-endoxifen in aromatase inhibitor-sensitive and aromatase inhibitor-resistant estrogen receptor-positive breast cancer
Source: Breast Cancer Res. 2020 May 19;22:51. doi: 10.1186/s13058-020-01286-7 (PMC7238733; doi:10.1186/s13058-020-01286-7)
Supplement: Supplementary file 4 — Additional file 4. The effects of the SERMs and fulvestrant on the growth of T47D, BT474, neo and HER2/18-expressing MCF7 cells in vitro. a-d Treatment of the cells with tamoxifen, Z-endoxifen, 4HT and fulvestrant in the presence of 1 nM E2 at the indicated concentrations for seven days. Growth was assessed by fixing the cells in glutaraldehyde followed by staining with crystal violet. Data is representative of six wells per treatment performed in biological triplicates and presented as mean ± SD. e Western blot analysis of HER2 and ERα expression in the indicated cell lines. Actin served as the loading control. Images are representative of three independent experiments. E2 = Estradiol. [file 13058_2020_1286_MOESM4_ESM.docx]

**
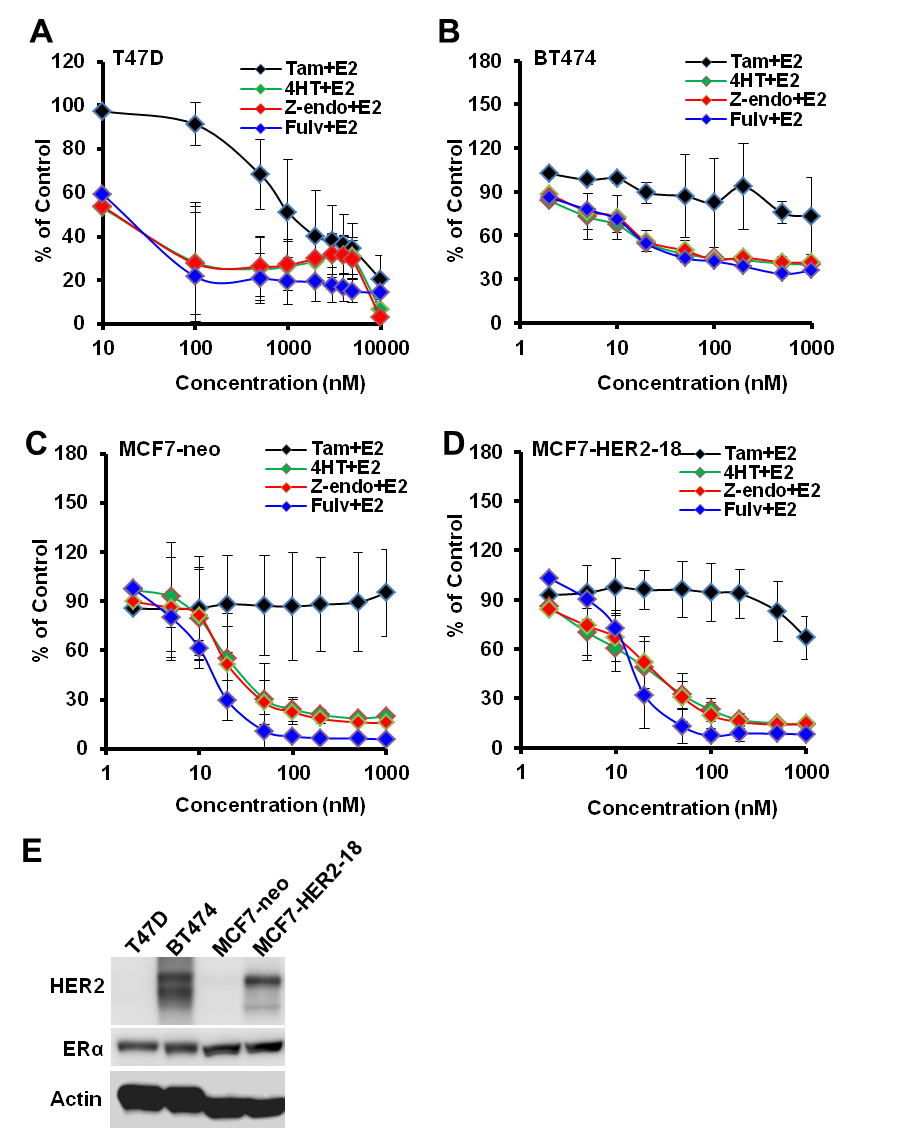
Additional file 4:**

**Figure S4. The effects of the SERMs and fulvestrant on the growth of T47D, BT474, neo and HER2/18-expressing MCF7 cells *in vitro***. **a-d** Treatment of the cells with tamoxifen, Z-endoxifen, 4HT and fulvestrant in the presence of 1 nM E2 at the indicated concentrations for seven days. Growth was assessed by fixing the cells in glutaraldehyde followed by staining with crystal violet. Data is representative of six wells per treatment performed in biological triplicates and presented as mean ± SD. **e** Western blot analysis of HER2 and ERα expression in the indicated cell lines. Actin served as the loading control. Images are representative of three independent experiments. E2 = Estradiol.
