# Supplementary material for: Antitumor activity of Z-endoxifen in aromatase inhibitor-sensitive and aromatase inhibitor-resistant estrogen receptor-positive breast cancer
Source: Breast Cancer Res. 2020 May 19;22:51. doi: 10.1186/s13058-020-01286-7 (PMC7238733; doi:10.1186/s13058-020-01286-7)
Supplement: Supplementary file 5 — Additional file 5. The effects of the SERMs on the growth of MDAMB231, MDAMB468 and BT20 cells in vitro. a Treatment of the cells with tamoxifen, Z-endoxifen and 4HT in the absence of estradiol for seven days. b Treatment with the aforementioned drugs in the presence of 1 nM E2 for seven days. Growth was assessed by fixing the cells in glutaraldehyde followed by staining with crystal violet. Data is representative of six wells per treatment performed in biological triplicates and presented as mean ± SD. E2 = Estradiol. [file 13058_2020_1286_MOESM5_ESM.docx]

**
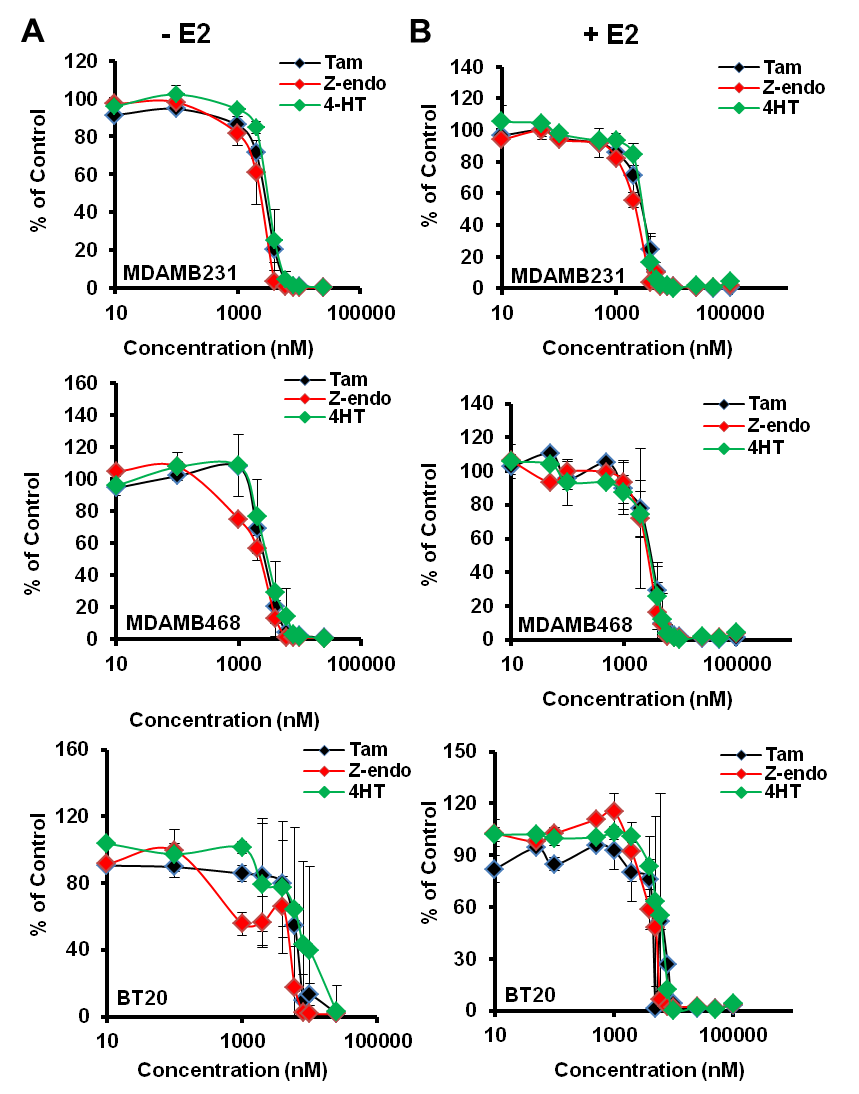
Additional file 5:**

**Figure S5. The effects of the SERMs on the growth of MDAMB231, MDAMB468 and BT20 cells *in vitro***. **a** Treatment of the cells with tamoxifen, Z-endoxifen and 4HT in the absence of estradiol for seven days. **b** Treatment with the aforementioned drugs in the presence of 1 nM E2 for seven days. Growth was assessed by fixing the cells in glutaraldehyde followed by staining with crystal violet. Data is representative of six wells per treatment performed in biological triplicates and presented as mean ± SD. E2 = Estradiol.
