# Supplementary material for: Antitumor activity of Z-endoxifen in aromatase inhibitor-sensitive and aromatase inhibitor-resistant estrogen receptor-positive breast cancer
Source: Breast Cancer Res. 2020 May 19;22:51. doi: 10.1186/s13058-020-01286-7 (PMC7238733; doi:10.1186/s13058-020-01286-7)
Supplement: Supplementary file 6 — Additional file 6. The effect of Z-endoxifen and tamoxifen on Ki67 protein in MCF7LR tumors. a Ki67 expression in letrozole (n = 3), Z-endoxifen (n = 5) or tamoxifen (n = 3) treated MCF7LR tumors analyzed by IHC. b Histogram of the percentage of Ki67 nuclear staining in these tumors. Differences in the gene expression in the SERM-treated MCF7LR tumors compared to the letrozole-treated MCF7LR tumors were compared using two-sample t-tests. Non-significant (ns), P > 0.05; **, P < 0.01; ***, P < 0.001 compared to Continued Letrozole treatment group. [file 13058_2020_1286_MOESM6_ESM.docx]

**
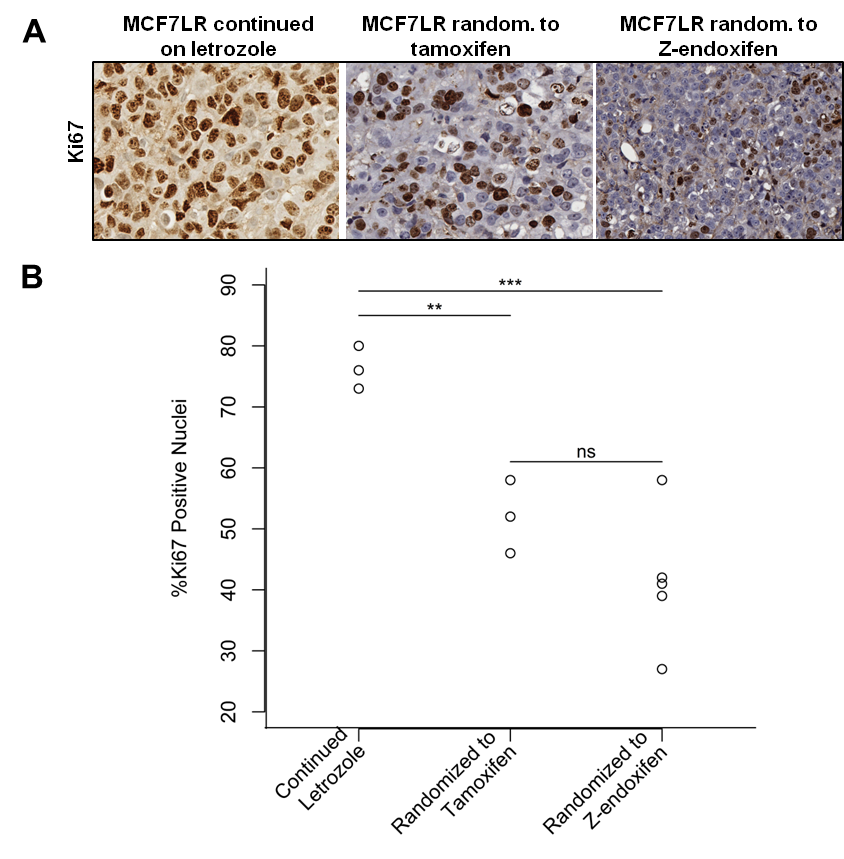
Additional file 6:**

**Figure S6. The effect of Z-endoxifen and tamoxifen on Ki67 protein in MCF7LR tumors**. **a** Ki67 expression in letrozole (n=3), Z-endoxifen (n=5) or tamoxifen (n=3) treated MCF7LR tumors analyzed by IHC. **b** Histogram of the percentage of Ki67 nuclear staining in these tumors. Differences in the gene expression in the SERM-treated MCF7LR tumors compared to the letrozole-treated MCF7LR tumors were compared using two-sample t-tests. Non-significant (ns), *P* > 0.05; **, *P* < 0.01; ***, *P* < 0.001 compared to Continued Letrozole treatment group.
