# Supplementary material for: Antitumor activity of Z-endoxifen in aromatase inhibitor-sensitive and aromatase inhibitor-resistant estrogen receptor-positive breast cancer
Source: Breast Cancer Res. 2020 May 19;22:51. doi: 10.1186/s13058-020-01286-7 (PMC7238733; doi:10.1186/s13058-020-01286-7)
Supplement: Supplementary file 12 — Additional file 12 Gene Set Enrichment Analysis (GSEA) of the estrogen signaling pathway. a The pathway enrichment score of the estrogene signaling pathway (from Kyoto Encyclopedia of Genes and Genome (KEGG)) in the treatment groups along with the nominal p-values and false discovery rate (FDR) q-values. b Enrichment plot of the KEGG estrogen signaling pathway of Z-endoxifen-treated MCF7LR tumors compared with letrozole-resistant MCF7LR tumors. The enrichment score and the p-value are listed. [file 13058_2020_1286_MOESM12_ESM.docx]

**
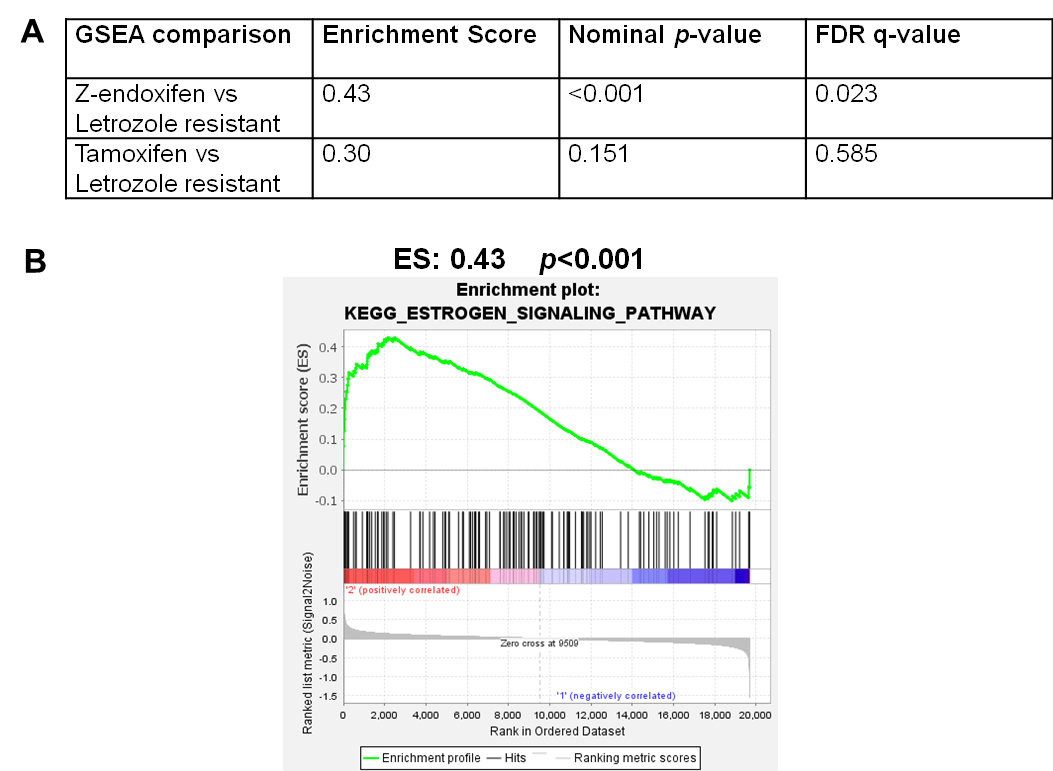
Additional file 12:**

**Figure S7. Gene Set Enrichment Analysis (GSEA) of the estrogen signaling pathway.** **a** The pathway enrichment score of the estrogene signaling pathway (from Kyoto Encyclopedia of Genes and Genome (KEGG)) in the treatment groups along with the nominal p-values and false discovery rate (FDR) q-values. **b** Enrichment plot of the KEGG estrogen signaling pathway of Z-endoxifen-treated MCF7LR tumors compared with letrozole-resistant MCF7LR tumors. The enrichment score and the *p*-value are listed.
