# Supplementary material for: Antitumor activity of Z-endoxifen in aromatase inhibitor-sensitive and aromatase inhibitor-resistant estrogen receptor-positive breast cancer
Source: Breast Cancer Res. 2020 May 19;22:51. doi: 10.1186/s13058-020-01286-7 (PMC7238733; doi:10.1186/s13058-020-01286-7)
Supplement: Supplementary file 13 — Additional file 13 Summary of Gene Set Variation Analysis (GSVA) for the estrogen signaling pathway. a The Table shows the enrichment score values of the estrogen signaling pathway for individual samples and the treatment groups. b The GSVA enrichment scores for the estrogen signaling pathway between the treatment groups. c Table shows the group mean value and p-value of the different comparisons. [file 13058_2020_1286_MOESM13_ESM.docx]

**
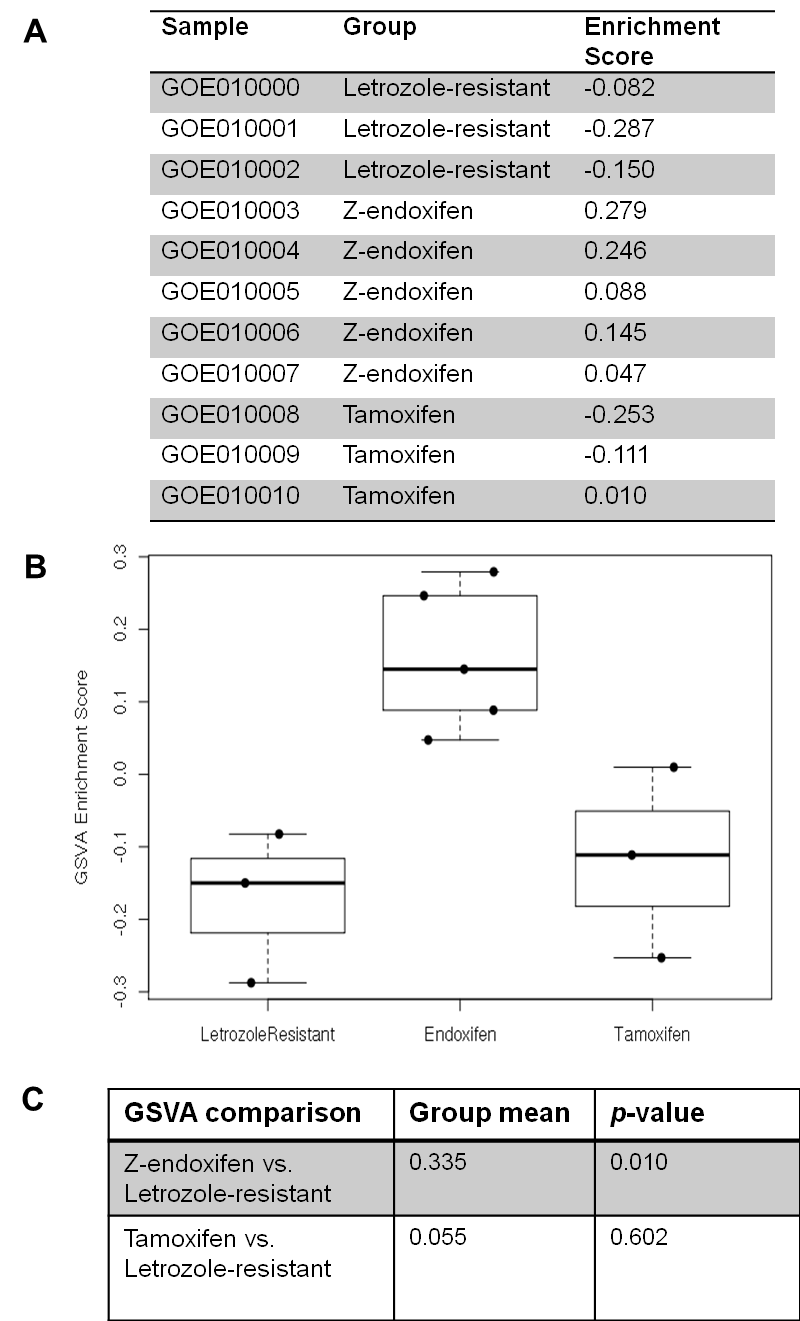
Additional file 13:**

**Figure S8: Summary of Gene Set Variation Analysis (GSVA) for the estrogen signaling pathway.** **a** The Table shows the enrichment score values of the estrogen signaling pathway for individual samples and the treatment groups. **b** The GSVA enrichment scores for the estrogen signaling pathway between the treatment groups. **c** Table shows the group mean value and *p*-value of the different comparisons.
